# Supplementary material for: DNA Binding and Anticancer Properties of New Pd(II)-Phosphorus Schiff Base Metal Complexes
Source: Pharmaceutics. 2022 Nov 8;14(11):2409. doi: 10.3390/pharmaceutics14112409 (PMC9697782; doi:10.3390/pharmaceutics14112409)
Supplement: Supplementary file 1 [file pharmaceutics-14-02409-s001.zip › pharmaceutics-1975014-supplementary.pdf]

SUPPLEMENTARY DATA

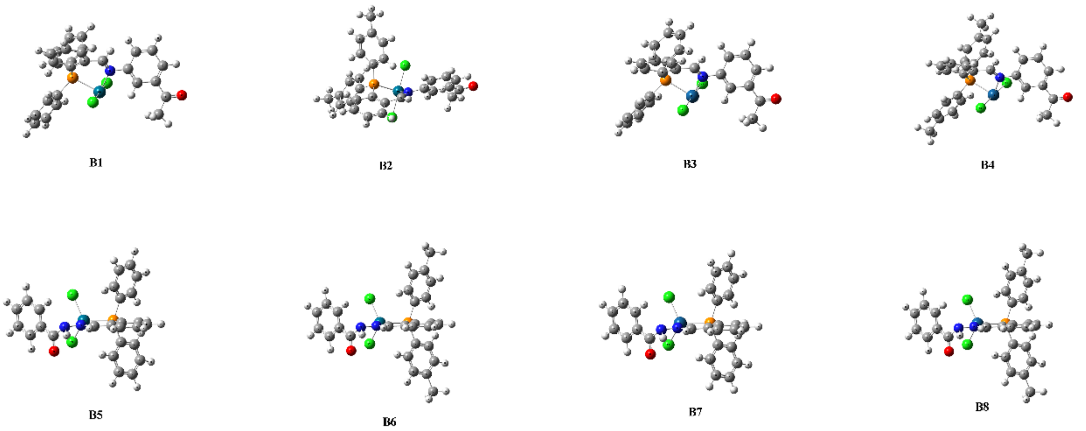

Figure S1. 3D structures of the optimized **B1**-**B8** complexes, respectively.

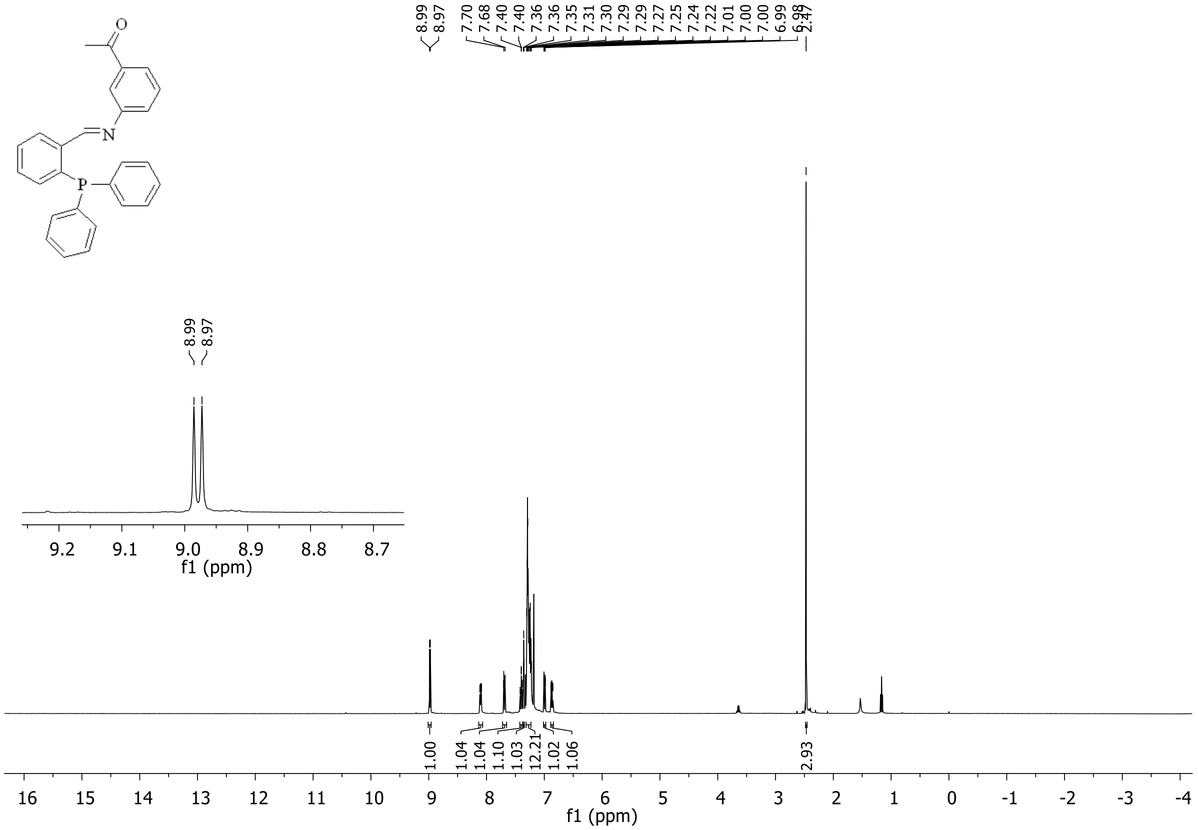

Figure S2. ^{1}H NMR spectra of compound **L1**

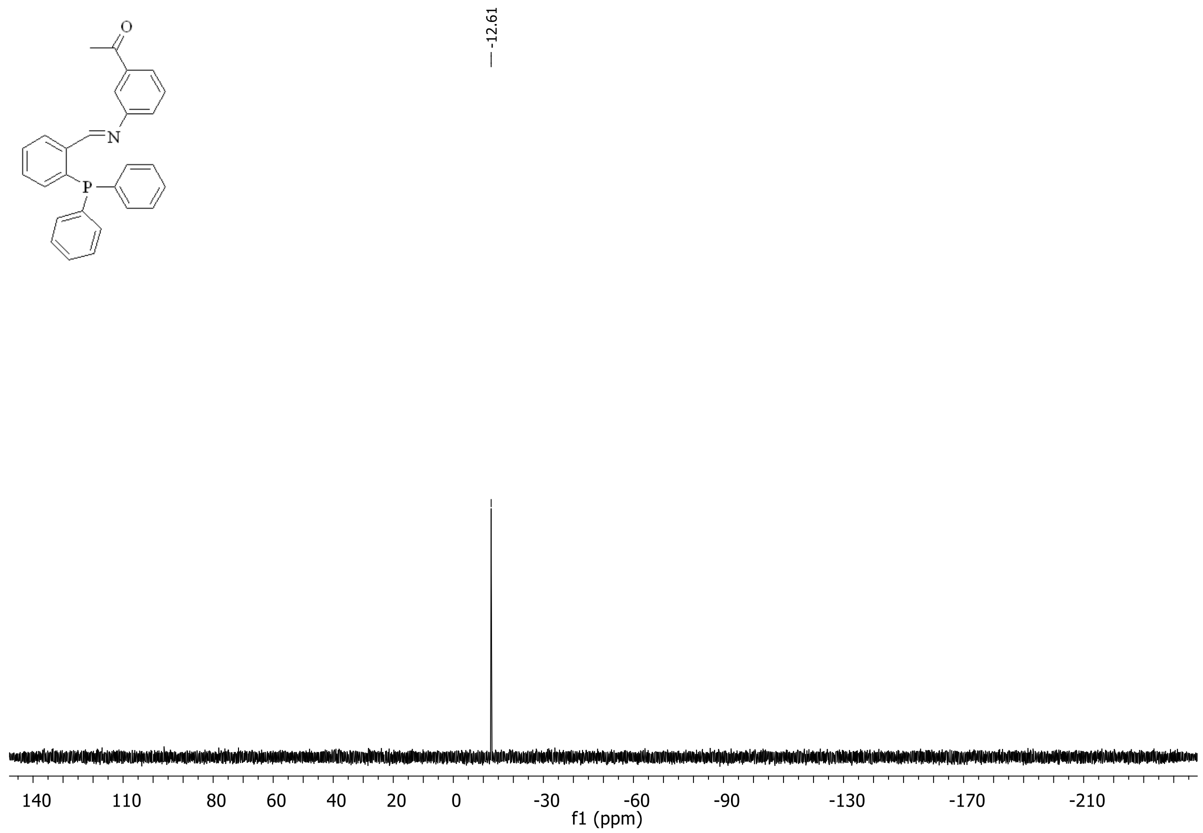

Figure S3. ^{31}P NMR spectra of compound **L1**

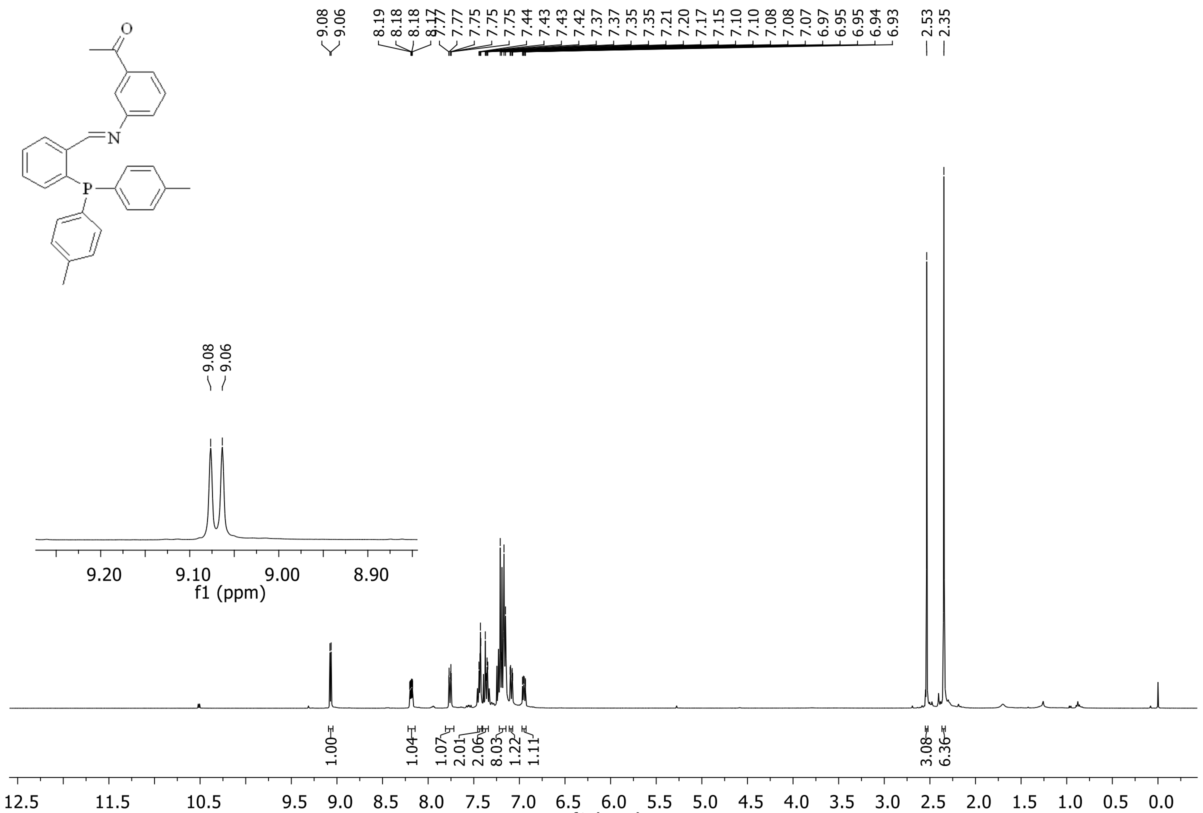

Figure S4. ^{1}H NMR spectra of compound **L2**

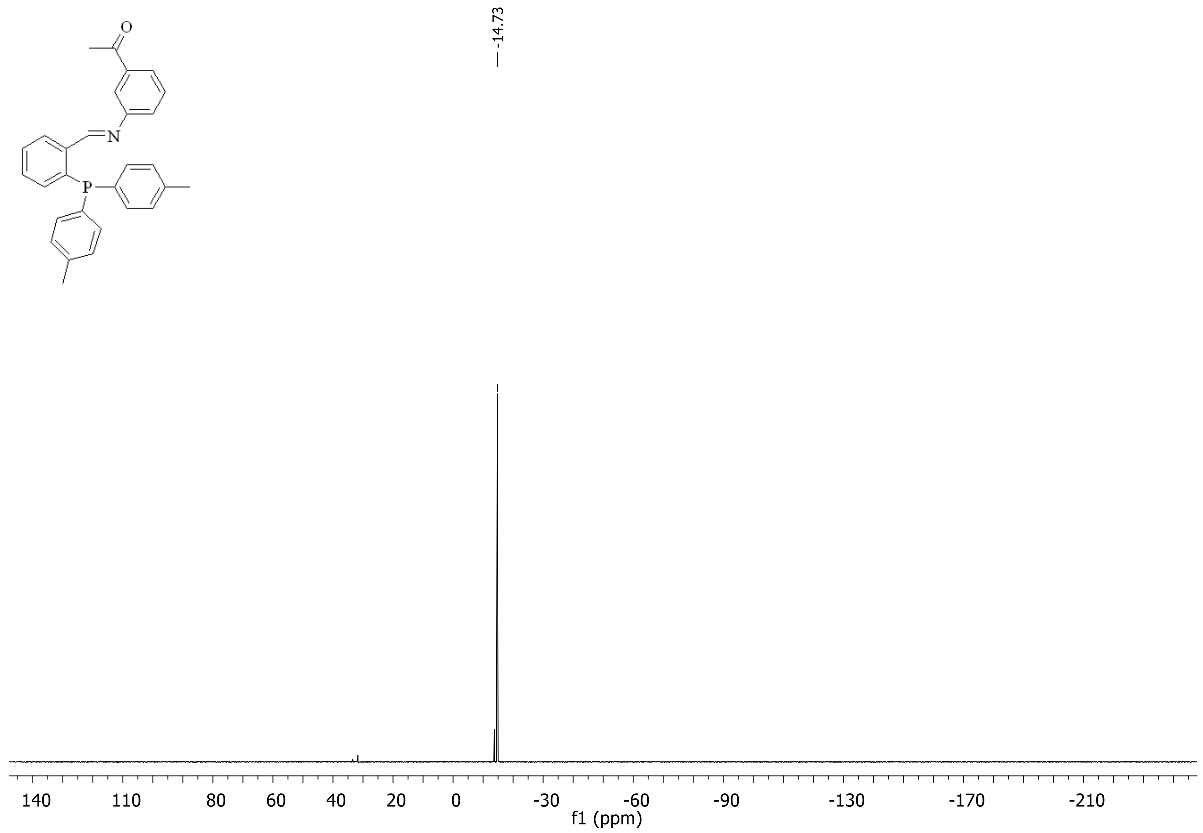

Figure S5. ^{31}P NMR spectra of compound **L2**

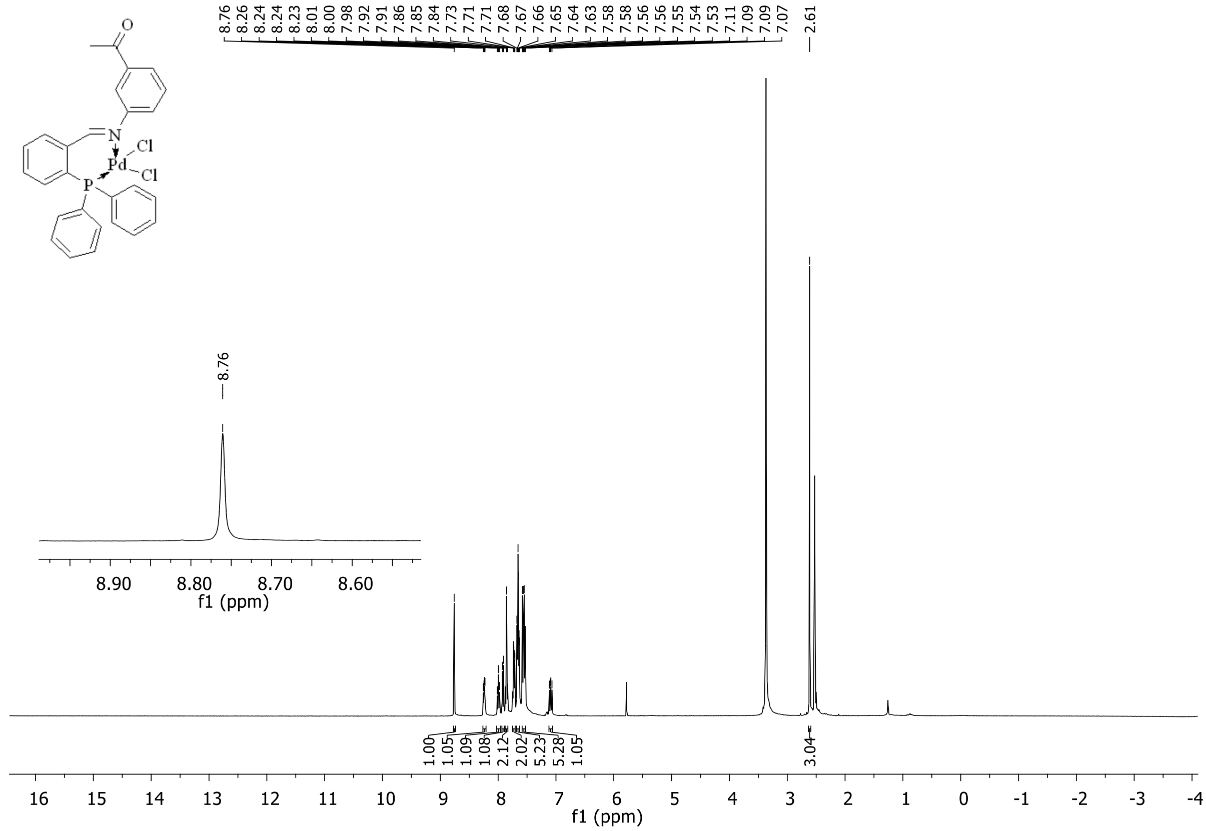

Figure S6. ^{1}H NMR spectra of compound **B1**

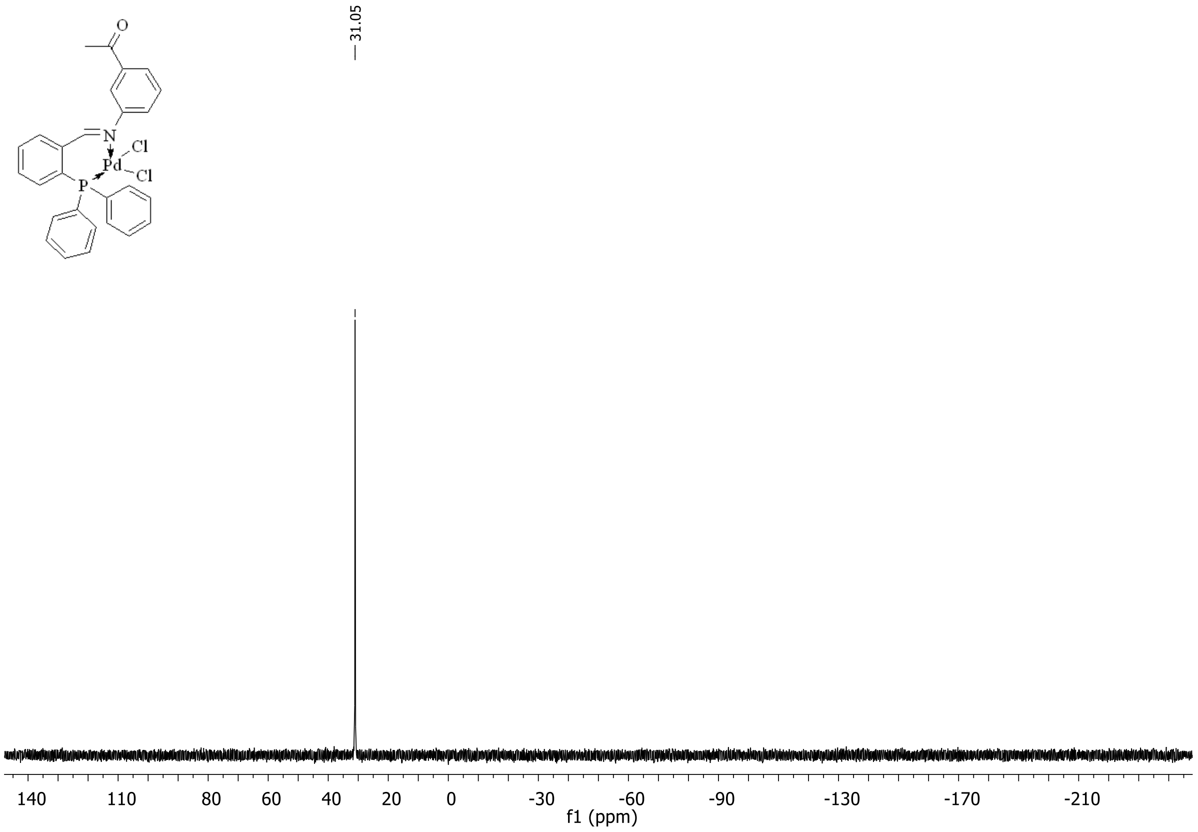

Figure S7. ^{31}P NMR spectra of compound **B1**

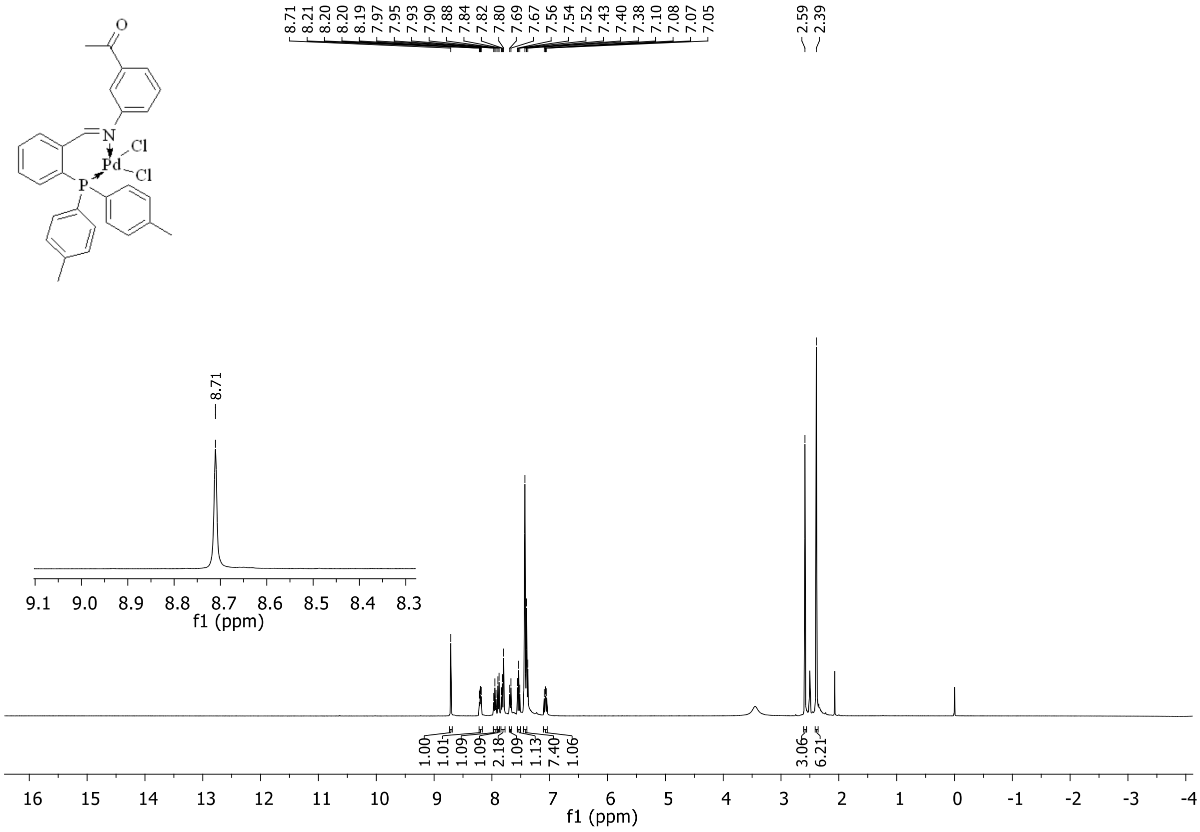

Figure S8. ^{1}H NMR spectra of compound **B2**

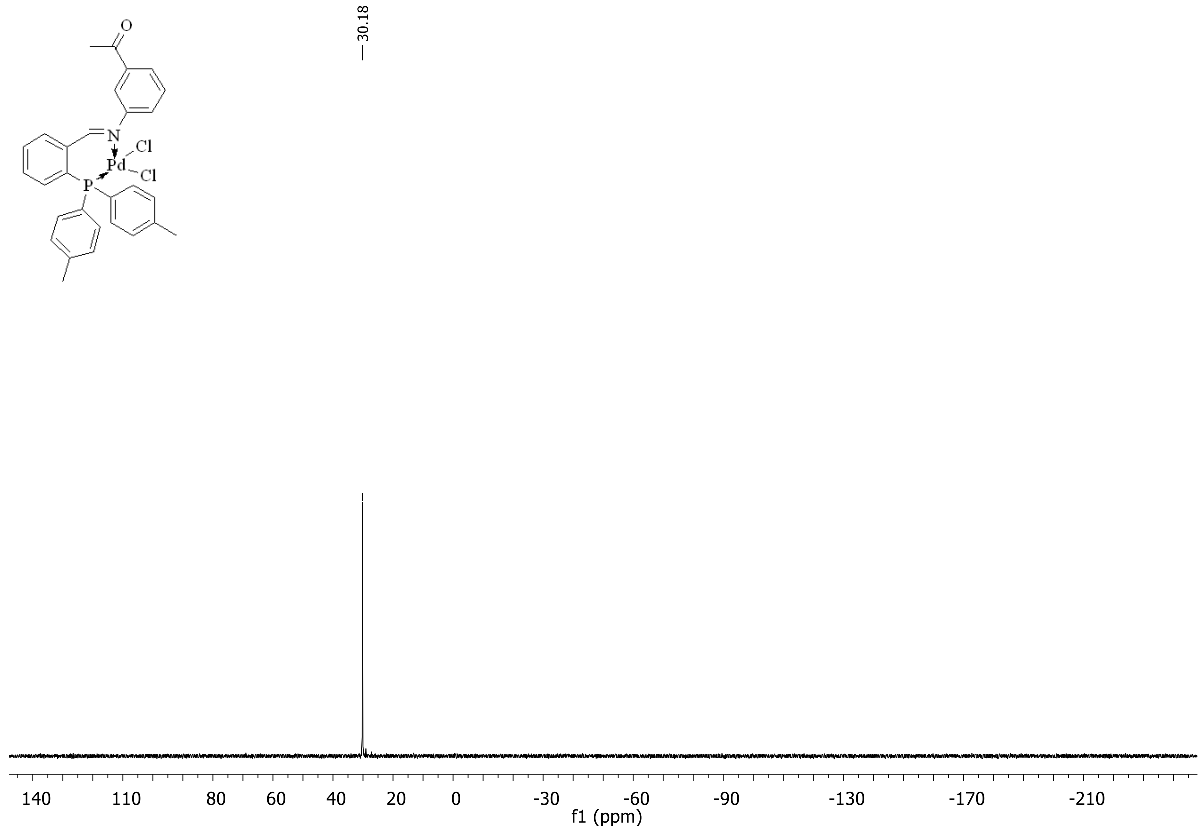

Figure S9. ^{31}P NMR spectra of compound **B2**

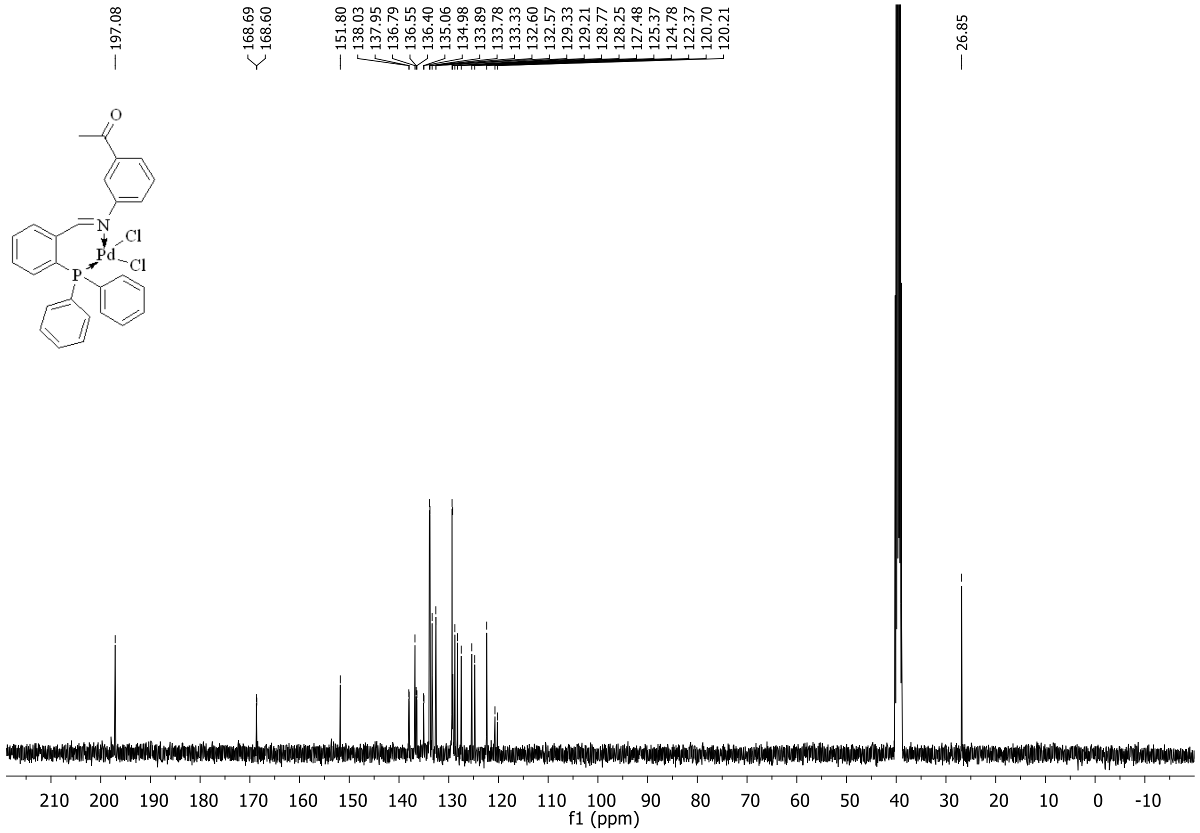

Figure S10. ^{13}C NMR spectra of compound B1

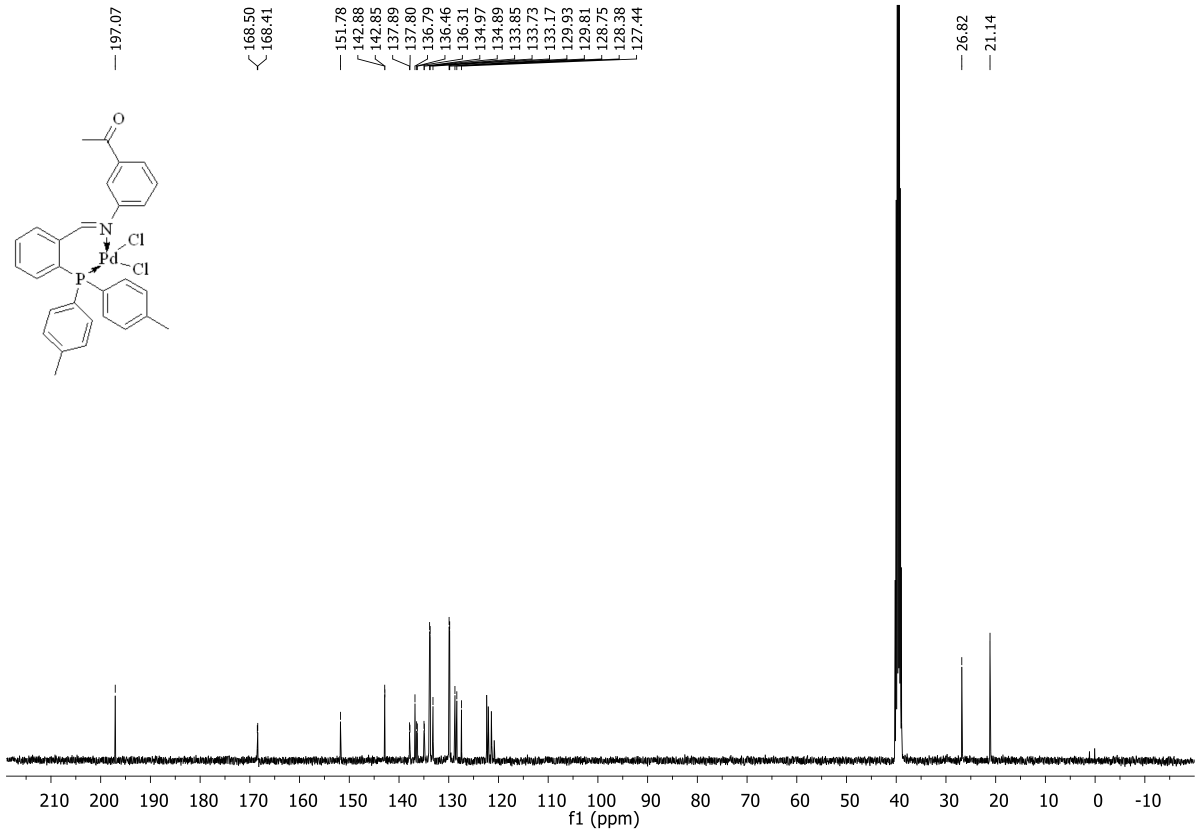

Figure S11. ^{31}C NMR spectra of compound B2

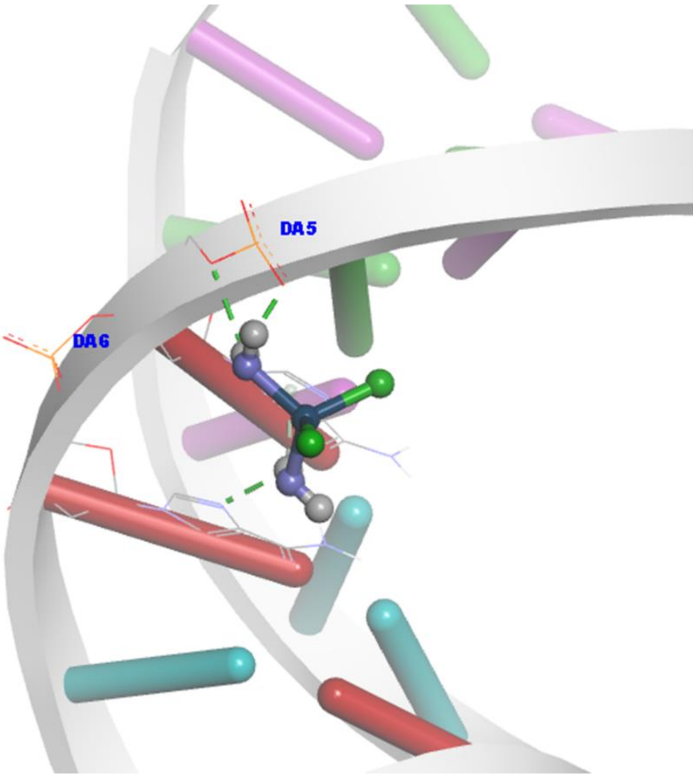

Figure S12. Docking conformation and interactions of cisplatin with DNA.

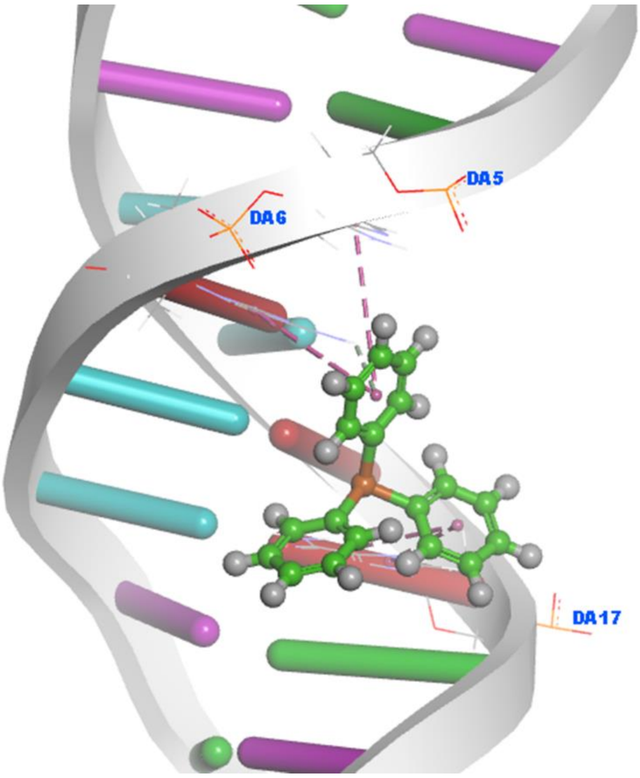

Figure S13. Docking conformation and interactions of triphenylphosphine with DNA.

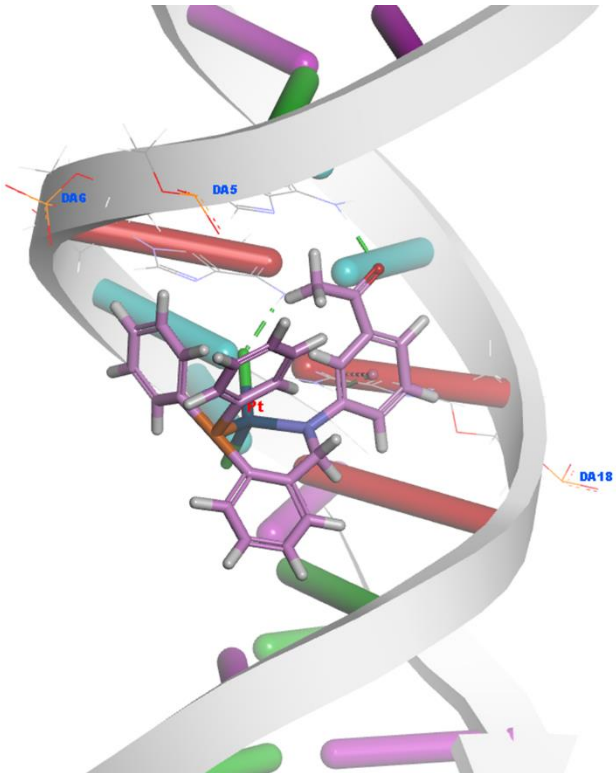

Figure S14. Docking conformation and interactions of the **B3** complex with DNA.

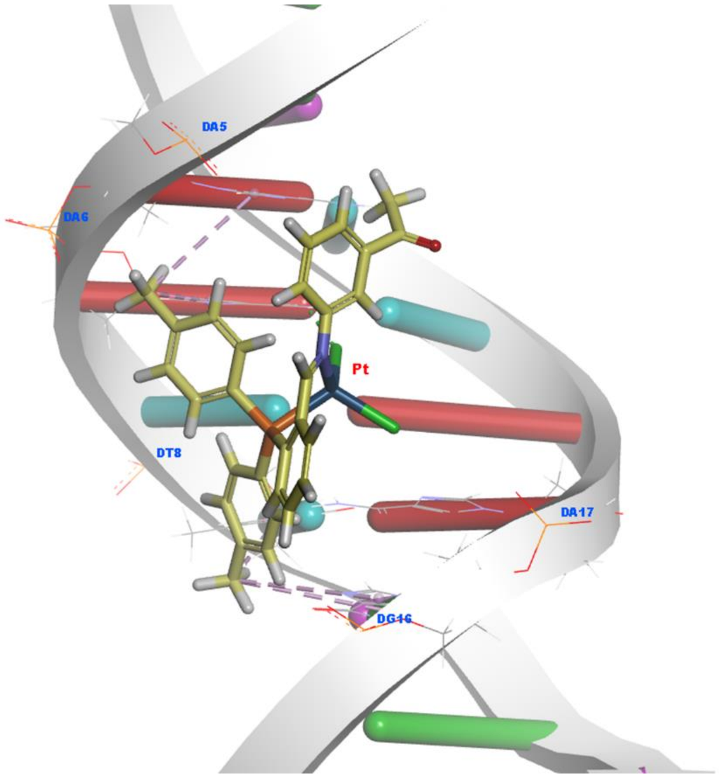

Figure S15. Docking conformation and interactions of the **B4** complex with DNA.

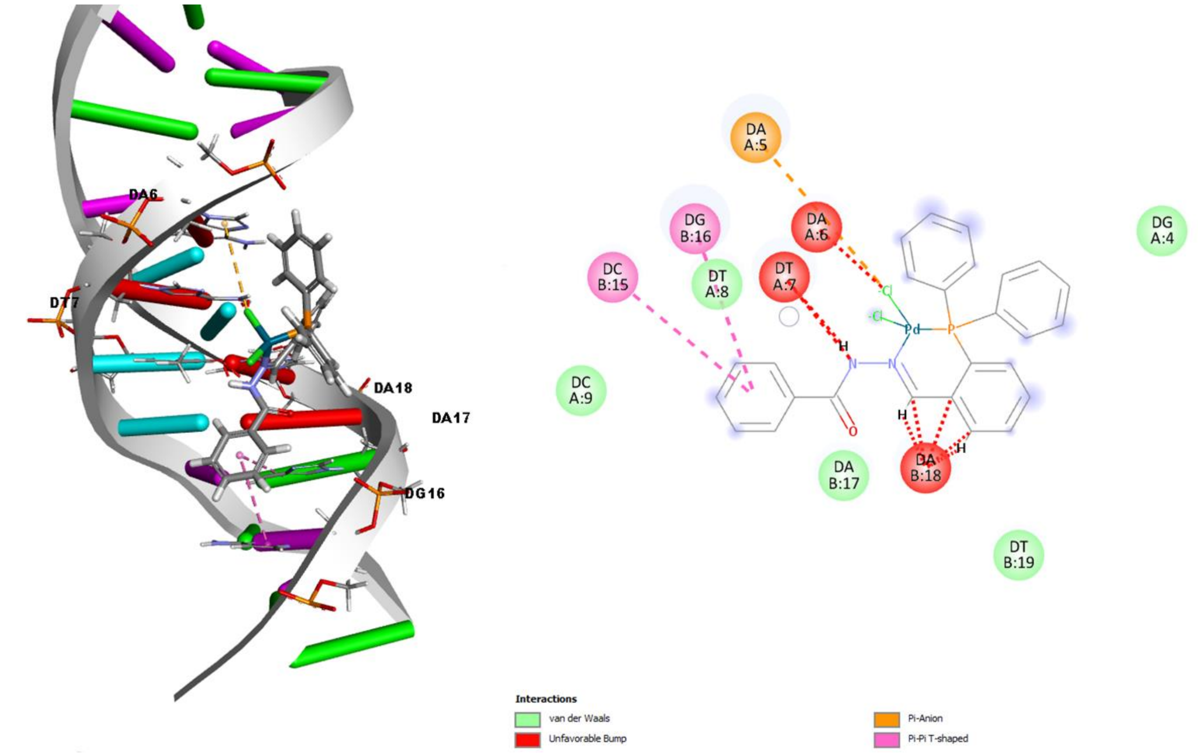

Figure S16. 3D and 2D docking interactions of the compound B5 with DNA.

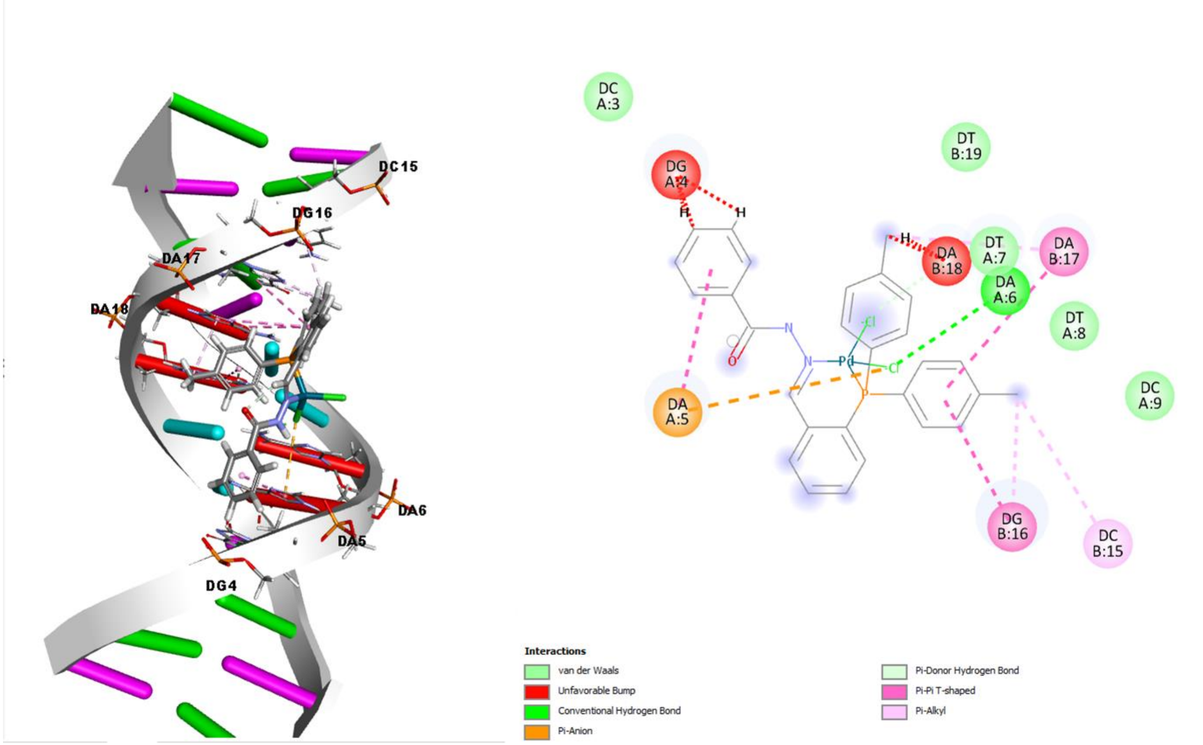

Figure S17. 3D and 2D docking interactions of the compound B6 with DNA.

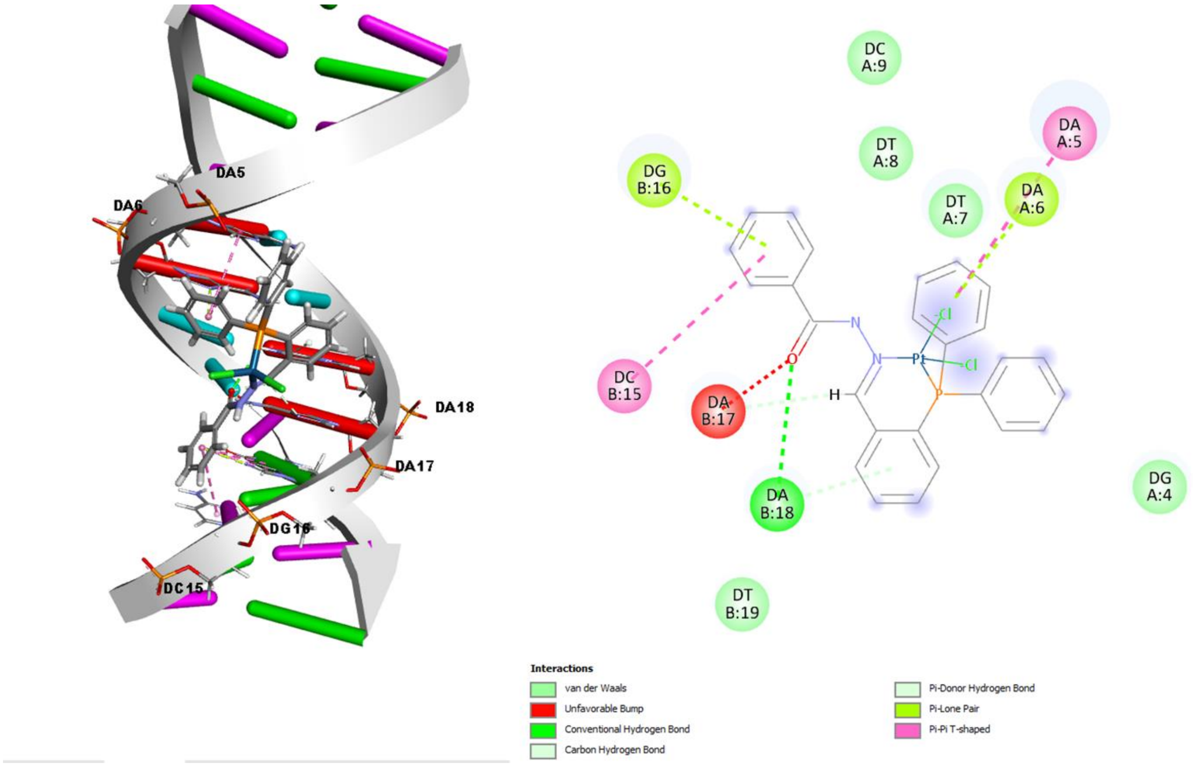

Figure S18. 3D and 2D docking interactions of the compound B7 with DNA.

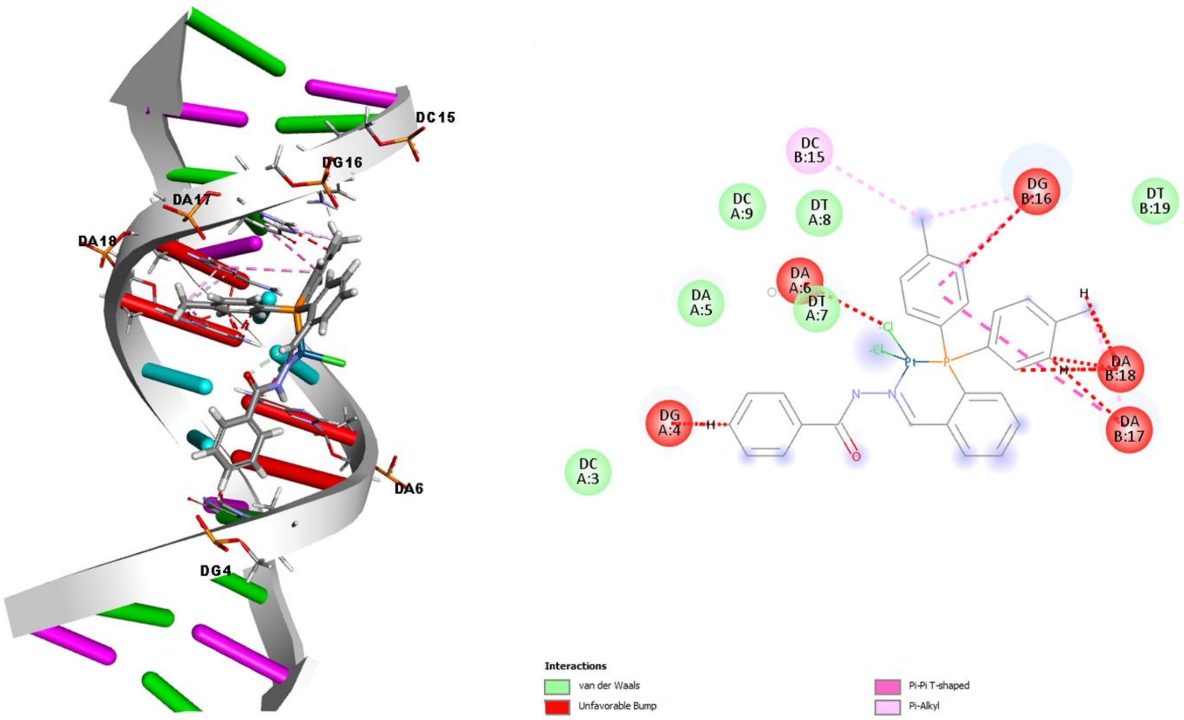

Figure S19. 3D and 2D docking interactions of the compound B8 with DNA.

Table S1. Interactions types and distances of the standard compounds (cisplatin: std-1 and triphenylphosphine: std-2) and the compound B1-B8 with DNA, respectively.

**DNA Interactions-std1**	**Distance Å**	**Bonding**	**Bonding Types**	**Binding site of target**	**Binding site of ligand**
:std1:H6 - A:DA5:O5'	2.6394	Hydrogen Bond	Conventional Hydrogen Bond	A:DA5:O5'	std1:H6
std1:H7 - A:DA5:OP2	1.6408	Hydrogen Bond	Conventional Hydrogen Bond	A:DA5:OP2	std1:H7
std1:H9 - A:DA6:N7	2.2295	Hydrogen Bond	Conventional Hydrogen Bond	A:DA6:N7	std1:H9
std1:H9 - A:DA5	2.7973	Hydrogen Bond	Pi-Donor Hydrogen Bond	A:DA5	std1:H9
**DNA Interactions-std2**	**Distance Å**	**Bonding**	**Bonding Types**	**Binding site of target**	**Binding site of ligand**
A:DA6:H61 - :std2	2.9341	Hydrogen Bond	Pi-Donor Hydrogen Bond	A:DA6:H61	std2
A:DA5 - :std2	5.4885	Hydrophobic	Pi-Pi T-shaped	A:DA5	std2
A:DA6 - :std2	4.7080	Hydrophobic	Pi-Pi T-shaped	A:DA6	std2
A:DA6 - :std2	5.5637	Hydrophobic	Pi-Pi T-shaped	A:DA6	std2
B:DA17 - :std2	4.3983	Hydrophobic	Pi-Pi T-shaped	B:DA17	std2
B:DA17 - :std2	5.8602	Hydrophobic	Pi-Pi T-shaped	B:DA17	std2
**DNA Interactions-B1**	**Distance Å**	**Bonding**	**Bonding Types**	**Binding site of target**	**Binding site of ligand**
A:DA6:H62 - :B1:O29	2.5790	Hydrogen Bond	Conventional Hydrogen Bond	A:DA6:H62	:B1:O29
B:DA18:H61 - :B1:O29	2.3822	Hydrogen Bond	Conventional Hydrogen Bond	B:DA18:H61	:B1:O29
B:DA17 - :B1	4.2083	Hydrophobic	Pi-Pi T-shaped	B:DA17	:B1
**DNA Interactions-B2**	**Distance Å**	**Bonding**	**Bonding Types**	**Binding site of target**	**Binding site of ligand**
B2:H5 - B:DA17:N7	1.6079	Hydrogen Bond	Carbon Hydrogen Bond	B:DA17:N7	B2:H5
A:DA5 -B2	4.5858	Hydrophobic	Pi-Pi Stacked	A:DA5	B2
A:DA5 - B2	5.4257	Hydrophobic	Pi-Pi T-shaped	A:DA5	B2
A:DT7 - B2	5.6535	Hydrophobic	Pi-Pi T-shaped	A:DT7	B2
B:DA17 -B2	4.5750	Hydrophobic	Pi-Pi T-shaped	B:DA17	B2
A:DG4 - B2:C35	4.8698	Hydrophobic	Pi-Alkyl	A:DG4	B2:C35

A:DA5 - B2:C34	5.1792	Hydrophobic	Pi-Alkyl	A:DA5	B2:C34
**DNA Interactions-B3**	**Distance Å**	**Bonding**	**Bonding Types**	**Binding site of target**	**Binding site of ligand**
A:DA5:H61 - B3:O29	1.9702	Hydrogen Bond	Conventional Hydrogen Bond	A:DA5:H61	B3:O29
A:DA6:H61 - B3:CL30	2.0322	Hydrogen Bond;Halogen	Conventional Hydrogen Bond;Halogen (Cl, Br, I)	A:DA6:H61	B3:CL30
B:DA18:H61 - B3	2.2287	Hydrogen Bond	Pi-Donor Hydrogen Bond	B:DA18:H61	B3
B:DA18 - B3	5.1754	Hydrophobic	Pi-Pi T-shaped	B:DA18	B3
**DNA Interactions-B4**	**Distance Å**	**Bonding**	**Bonding Types**	**Binding site of target**	**Binding site of ligand**
A:DA6:H61 -B4:CL30	2.0434	Hydrogen Bond;Halogen	Conventional Hydrogen Bond;Halogen (Cl, Br, I)	A:DA6:H61	B4:CL30
A:DA5 -B4:C35	4.9879	Hydrophobic	Pi-Alkyl	A:DA5	B4:C35
A:DA6 -B4:C35	4.1503	Hydrophobic	Pi-Alkyl	A:DA6	B4:C35
A:DT8 -B4:C34	5.1468	Hydrophobic	Pi-Alkyl	A:DT8	B4:C34
B:DG16 -B4:C34	5.1358	Hydrophobic	Pi-Alkyl	B:DG16	B4:C34
B:DG16 -B4:C34	5.2304	Hydrophobic	Pi-Alkyl	B:DG16	B4:C34
**DNA Interactions-B5**	**Distance Å**	**Bonding**	**Bonding Types**	**Binding site of target**	**Binding site of ligand**
:B5:CL33 - A:DA5	4.7311	Electrostatic	Pi-Anion	:B5:CL33	A:DA5
B:DC15 - :B5	5.2471	Hydrophobic	Pi-Pi T-shaped	B:DC15	:B5
B:DG16 - :B5	5.2013	Hydrophobic	Pi-Pi T-shaped	B:DG16	:B5
A:DA6:N6 - :B5:CL33	2.1650	Unfavorable	Unfavorable Bump	A:DA6:N6	:B5:CL33
A:DA6:H61 - :B5:CL33	1.3547	Unfavorable	Unfavorable Bump;Conventional Hydrogen Bond;Halogen (Cl, Br, I)	A:DA6:H61	:B5:CL33
A:DT7:O4 - :B5:N8	2.1142	Unfavorable	Unfavorable Bump	A:DT7:O4	:B5:N8
B:DA18:N7 - :B5:H8	1.4712	Unfavorable	Unfavorable Bump	B:DA18:N7	:B5:H8
B:DA18:N6 - :B5:C11	1.6515	Unfavorable	Unfavorable Bump	B:DA18:N6	:B5:C11
B:DA18:N6 - :B5:C12	1.9633	Unfavorable	Unfavorable Bump	B:DA18:N6	:B5:C12

B:DA18:N6 - :B5:C17	2.1790	Unfavorable	Unfavorable Bump	B:DA18:N6	:B5:C17
B:DA18:N6 - :B5:H7	1.7185	Unfavorable	Unfavorable Bump	B:DA18:N6	:B5:H7
B:DA18:H61 - :B5:C11	1.5162	Unfavorable	Unfavorable Bump	B:DA18:H61	:B5:C11
B:DA18:H61 - :B5:C12	1.0206	Unfavorable	Unfavorable Bump	B:DA18:H61	:B5:C12
B:DA18:H61 - :B5:C17	1.2181	Unfavorable	Unfavorable Bump	B:DA18:H61	:B5:C17
B:DA18:H61 - :B5:H8	1.4681	Unfavorable	Unfavorable Bump	B:DA18:H61	:B5:H8
B:DA18:H62 - :B5:C11	1.6637	Unfavorable	Unfavorable Bump	B:DA18:H62	:B5:C11
:B5:H6 - A:DT7:O4	1.4794	Unfavorable	Unfavorable Bump;Conventional Hydrogen Bond	:B5:H6	A:DT7:O4
**DNA Interactions-B6**	**Distance Å**	**Bonding**	**Bonding Types**	**Binding site of target**	**Binding site of ligand**
A:DA6:H61 - :B6:CL33	1.9407	Hydrogen Bond;Halogen	Conventional Hydrogen Bond;Halogen (Cl, Br, I)	A:DA6:H61	:B6:CL33
:B6:H7 - :B6:O10	2.2210	Hydrogen Bond	Carbon Hydrogen Bond	:B6:H7	:B6:O10
:B6:CL33 - A:DA5	4.6273	Electrostatic	Pi-Anion	A:DA5	:B6:CL33
B:DA18:H61 - :B6	1.7053	Hydrogen Bond	Pi-Donor Hydrogen Bond	B:DA18:H61	:B6
A:DA5 - :B6	4.8547	Hydrophobic	Pi-Pi T-shaped	A:DA5	:B6
B:DG16 - :B6	5.1974	Hydrophobic	Pi-Pi T-shaped	B:DG16	:B6
B:DA17 - :B6	5.7331	Hydrophobic	Pi-Pi T-shaped	B:DA17	:B6
B:DA17 - :B6	5.6734	Hydrophobic	Pi-Pi T-shaped	B:DA17	:B6
B:DA18 - :B6	4.9981	Hydrophobic	Pi-Pi T-shaped	B:DA18	:B6
B:DC15 - :B6:C35	4.4823	Hydrophobic	Pi-Alkyl	B:DC15	:B6:C35
B:DG16 - :B6:C35	4.4751	Hydrophobic	Pi-Alkyl	B:DG16	:B6:C35
B:DG16 - :B6:C35	4.8113	Hydrophobic	Pi-Alkyl	B:DG16	:B6:C35
B:DA17 - :B6:C34	4.3797	Hydrophobic	Pi-Alkyl	B:DA17	:B6:C34
B:DA18 - :B6:C34	4.7311	Hydrophobic	Pi-Alkyl	B:DA18	:B6:C34
A:DG4:C8 - :B6:H2	1.6618	Unfavorable	Unfavorable Bump	A:DG4:C8	:B6:H2

A:DG4:H2'1 - :B6:H1	1.3727	Unfavorable	Unfavorable Bump	A:DG4:H2'1	:B6:H1
A:DG4:H8 - :B6:C2	1.6612	Unfavorable	Unfavorable Bump	A:DG4:H8	:B6:C2
A:DG4:H8 - :B6:H2	0.6467	Unfavorable	Unfavorable Bump	A:DG4:H8	:B6:H2
B:DA18:N7 - :B6:C34	2.2334	Unfavorable	Unfavorable Bump	B:DA18:N7	:B6:C34
B:DA18:N7 - :B6:H24	1.7305	Unfavorable	Unfavorable Bump	B:DA18:N7	:B6:H24
**DNA Interactions-B7**	**Distance Å**	**Bonding**	**Bonding Types**	**Binding site of target**	**Binding site of ligand**
B:DA18:H62 - B7::O10	3.0998	Hydrogen Bond	Conventional Hydrogen Bond	B:DA18:H62	B7::O10
B7::H7 - B:DA17:N7	2.7335	Hydrogen Bond	Carbon Hydrogen Bond	B7::H7	B:DA17:N7
B7::H7 - B7::O10	2.2638	Hydrogen Bond	Carbon Hydrogen Bond	B7::H7	B7::O10
B:DA18:H61 - B7:	2.4236	Hydrogen Bond	Pi-Donor Hydrogen Bond	B:DA18:H61	B7:
A:DA6:N7 - B7:	2.6399	Other	Pi-Lone Pair	A:DA6:N7	B7:
B:DG16:N7 - B7:	2.7502	Other	Pi-Lone Pair	B:DG16:N7	B7:
A:DA5 - B7:	5.0104	Hydrophobic	Pi-Pi T-shaped	A:DA5	B7:
A:DA6 - B7:	5.0897	Hydrophobic	Pi-Pi T-shaped	A:DA6	B7:
B:DC15 - B7:	4.9911	Hydrophobic	Pi-Pi T-shaped	B:DC15	B7:
B:DG16 - B7:	3.9387	Hydrophobic	Pi-Pi T-shaped	B:DG16	B7:
B:DG16 - B7:	4.9909	Hydrophobic	Pi-Pi T-shaped	B:DG16	B7:
B:DA17:H61 - B7:O10	1.5283	Unfavorable	Unfavorable Bump	B:DA17:H61	B7:O10
**DNA Interactions-B8**	**Distance Å**	**Bonding**	**Bonding Types**	**Binding site of target**	**Binding site of ligand**
:B8:H7 - :B8:O10	2.1993	Hydrogen Bond	Carbon Hydrogen Bond	:B8:H7	:B8:O10
B:DG16 - :B8	4.7999	Hydrophobic	Pi-Pi T-shaped	B:DG16	:B8
B:DA17 - :B8	5.6722	Hydrophobic	Pi-Pi T-shaped	B:DA17	:B8
B:DC15 - :B8:C34	3.7848	Hydrophobic	Pi-Alkyl	B:DC15	:B8:C34
B:DG16 - :B8:C34	4.1940	Hydrophobic	Pi-Alkyl	B:DG16	:B8:C34
B:DG16 - :B8:C34	4.7479	Hydrophobic	Pi-Alkyl	B:DG16	:B8:C34

B:DA17 - :B8:C33	3.3116	Hydrophobic	Pi-Alkyl	B:DA17	:B8:C33
B:DA17 - :B8:C33	4.7411	Hydrophobic	Pi-Alkyl	B:DA17	:B8:C33
B:DA18 - :B8:C33	4.8834	Hydrophobic	Pi-Alkyl	B:DA18	:B8:C33
A:DG4:C8 - :B8:H2	1.6368	Unfavorable	Unfavorable Bump	A:DG4:C8	:B8:H2
A:DG4:H8 - :B8:C2	1.7474	Unfavorable	Unfavorable Bump	A:DG4:H8	:B8:C2
A:DG4:H8 - :B8:H2	0.6903	Unfavorable	Unfavorable Bump	A:DG4:H8	:B8:H2
A:DA6:H61 - :B8:CL32	1.7167	Unfavorable	Unfavorable Bump;Conventional Hydrogen Bond;Halogen (Cl, Br, I)	A:DA6:H61	:B8:CL32
B:DG16:N7 - :B8:C22	2.2656	Unfavorable	Unfavorable Bump	B:DG16:N7	:B8:C22
B:DA17:N7 - :B8:C29	2.2157	Unfavorable	Unfavorable Bump	B:DA17:N7	:B8:C29
B:DA18:N7 - :B8:C33	2.2604	Unfavorable	Unfavorable Bump	B:DA18:N7	:B8:C33
B:DA18:N7 - :B8:H21	1.6330	Unfavorable	Unfavorable Bump	B:DA18:N7	:B8:H21
B:DA18:N7 - :B8:H22	1.3718	Unfavorable	Unfavorable Bump	B:DA18:N7	:B8:H22
B:DA18:N6 - :B8:C29	2.2730	Unfavorable	Unfavorable Bump	B:DA18:N6	:B8:C29
B:DA18:N6 - :B8:C30	2.2011	Unfavorable	Unfavorable Bump	B:DA18:N6	:B8:C30
B:DA18:H61 - :B8:C29	1.5769	Unfavorable	Unfavorable Bump	B:DA18:H61	:B8:C29
B:DA18:H61 - :B8:C30	1.6737	Unfavorable	Unfavorable Bump	B:DA18:H61	:B8:C30
